# Causes of Blindness in the Adult Population in Somalia

**DOI:** 10.4274/tjo.galenos.2020.82504

**Published:** 2020-10-30

**Authors:** Mustafa Kalaycı

**Affiliations:** 1 Somali Mogadishu-Turkey Recep Tayyip Erdogan Training and Research Hospital, Clinic of Ophthalmology, Mogadishu, Somalia

**Keywords:** Blindness, World Health Organization, Somalia, trauma, cataract

## Abstract

**Objectives:**

To evaluate the causes and frequency of blindness among the adult Somali population according to the World Health Organization (WHO) criteria.

**Materials and Methods:**

The data of 2,605 patients over 18 years old who presented to our tertiary hospital in Mogadishu (the capital of Somalia) were evaluated. Patients with best corrected visual acuity of less than 3/60 in both eyes were categorized as bilaterally blind and those with best corrected visual acuity of less than 3/60 in one eye but 3/60 or better in the other eye were classified as monocularly blind, as per the WHO classification.

**Results:**

Of 2,605 patients, 1,251 (48%) were female and 1,354 (52%) were male. Among these, 256 patients were determined to have blindness in one or both eyes and were included in the study. The patients ranged in age from 19 to 85, and the mean age was 52.4±14.6 years. The overall blindness rate in the Somali population was 9.8%. In the monocularly blind group, the most common factor was trauma complication (23.6%), followed by cataract (19%) and diabetic retinopathy (13.2%). In the bilaterally blind group, the most common factors were cataract (26.9%), diabetic retinopathy (21.1%), and glaucoma (15.4%).

**Conclusion:**

Trauma is the leading cause of blindness due to the security conditions in the country. Establishing and increasing the number of free public health centers in Somalia can reduce the frequency of blindness.

## Introduction

Blindness is an important public health problem with economic and social dimensions. Blindness and low vision are indicators of the general health status of a society. Globally, 285 million people suffer from visual impairment, 39 million of whom have a level of vision below the threshold for blindness.^1^ Approximately 90% of blind people live in developing countries.^2^ In addition, 80% of blindness occurs due to preventable or treatable causes. The most common cause of blindness worldwide is diabetic retinopathy.^3^ Diabetes is a common disease worldwide that causes ocular damage, including diabetic retinopathy.

The prevalence and causes of blindness and low vision vary in different societies based on their level of development. According to data from the World Health Organization (WHO), the prevalence of blindness is 7.3/1,000 in Africa, 3.5/1,000 in America, 8.5/1,000 in the Eastern Mediterranean Region, and 3.0/1,000 in Europe.^4^

The appropriate allocation of resources is important for effective delivery of health services in underdeveloped and developing countries. In this respect, data on the causes of vision loss may be helpful in planning and utilizing resources appropriately. To be able to prevent blindness, appropriate data must first be collected.

Somalia is an underdeveloped country in Sub-Saharan Africa with a population of 14 million. To date, there has been no study on the prevalence of blindness in Somalia.

This hospital-based study was conducted at Somalia Mogadishu-Turkey Recep Tayyip Erdogan Training and Research Hospital and aimed to evaluate the causes and frequency of blindness among Somali adults.

## Materials and Methods

### Patient Data

The files of 2605 patients over 18 years of age who presented to the Somalia Mogadishu-Turkey Recep Tayyip Erdogan Training and Research Hospital, Clinic of Eye between January 2019 and January 2020 were analyzed retrospectively. Of these, 256 patients determined to have monocular or bilateral blindness were included in the study. Approval for the study was obtained from the Somalia Mogadishu-Turkey Recep Tayyip Erdogan Education and Research Hospital Local Ethics Committee.

The demographic data and medical and family histories of all patients were recorded. Best corrected visual acuity (BCVA) was evaluated using a standard Snellen chart. Intraocular pressure (IOP) was measured using a pneumatic tonometer. Values 21 mmHg or higher with the pneumatic tonometer were measured again with a Goldmann applanation tonometer. Anterior segment examinations were performed with slit-lamp biomicroscope. Detailed dilated fundus examinations were performed and when necessary, tests and imaging such as computerized visual field test, optical coherence tomography, ocular ultrasonography, magnetic resonance imaging (MRI), computed tomography, blood tests, fasting blood glucose, and hemoglobin A1C were requested.

According to the WHO criteria, patients with BCVA worse than 3/60 in both eyes were classified as bilaterally blind while patients with BCVA worse than 3/60 in one eye and better than 3/60 in the fellow eye were classified as monocularly blind.^5^

In most cases, blindness was associated with a single cause. In cases with more than one cause, the factor with the greatest impact in the development of blindness was selected as the main cause, in accordance with the WHO recommendation.^6^

### Statistical Analysis

Statistical analyses were performed using IBM SPSS Statistics version 23.0 software (IBM Corp., Armonk, NY, USA). Mean, standard deviation, and minimum-maximum values were determined for continuous variables. Frequency analysis was performed.

## Results

Of the 256 patients, 120 (46.8%) were women and 136 (53.2%) were men. The patients were between 19 and 85 years of age, with a mean age of 52.4±14.6 years. Both monocular and bilateral blindness increased with age (Table 1). This finding was statistically significant (p<0.05).

In this hospital-centered study, the overall blindness rate was found to be 9.8% in the adult Somali population. The number of blind women was 120 (8%) and the number of blind men was 136 (10%). Blindness was bilateral in 104 patients (4%) and monocular in 152 patients (5.8%).

In the monocular blindness group, the most common cause was trauma complication (n=36, 23.6%), followed by cataract (n=29, 19%) and diabetic retinopathy (20, 13.2%) (Table 2).

For bilateral blindness, the most common causes were cataract (n=28, 26.9%), diabetic retinopathy (n=22, 21.1%), and glaucoma (n=16, 15.4%) (Table 3).

In the bilateral blindness group, vision loss was caused by retinitis pigmentosa in 2 patients (1.9%) and degenerative myopia in 2 patients (1.9%), while in the monocular group, the cause of vision loss was deep amblyopia in 3 patients (2%) and pterygium in 2 patients (1.4%).

## Discussion

The prevalence of blindness among adults was determined to be 9.8% in this study. This rate was reported as 7.3% in Ethiopia, 5.8% in Mali, 13.7% in Jordan, 10.9% in South Africa, and 7.8% in Kenya, and may vary between countries based on socioeconomic level, ethnic diversity, the healthcare system, the number and quality of eye care professionals, as well as whether or not there are institutions that support them.^7,8,9,10^

In our study, it was found that the prevalence of blindness increased with age. The higher frequency of glaucoma, cataract, and age-related retinal diseases in adults aged 60 and older may explain the higher rate of blindness in this group. Our findings were similar to those in other countries.^11,12,13^

Trauma complications were found to be the most common cause of monocular blindness. This may be a result of the terrorist acts and state of chaos that have persisted in the country for years. Based on our observations, this high rate may be due to the frequent bomb explosions in Somalia (twice a month on average) and the fact that nearly everyone owns a weapon and attempts to settle even minor arguments with firearms or non-firearm weapons. It was also observed in this study that trauma was more common among men. In Somalia, the fact that men are more active and work outside make them vulnerable to trauma. Similar findings were reported in a study related to ocular trauma patterns conducted in Nepal and a study conducted in the Bursa region of Turkey by Argun Kivanc et al.^14,15^ Both studies showed that the agriculture and construction sectors generally employ men and that ocular trauma is more common among workers in these sectors.^15^ Most of the male patients in our study group were also working in these areas, and our findings were similar to those reported in these two studies.

Furthermore, the majority of the Somalian population lives in rural areas and earns a living from agriculture and animal husbandry. Because patients also pay their own healthcare costs, it has been observed that early presentation to health services after ocular trauma is uncommon among agricultural workers. This seems to be another reason for the high rate of trauma-related blindness.

As ours is the only tertiary care hospital in Somalia, the country’s complicated ophthalmological cases are referred to our clinic. It has been observed that patients living far from the capital who present to local doctors after ocular trauma are referred to our clinic for surgical treatment. Referred patients are able to present to our clinic a few days later. No matter how quickly these patients are treated in our clinic, their vision may remain below the blindness threshold because of late presentation.

According to WHO data, the leading cause of blindness worldwide is cataract (51%).^6^ Blindness is defined as having a level of vision not exceeding 3/60 despite all treatments. Today, however, cataract is a surgically treatable condition. WHO’s inclusion of cataract patients in the classification of blindness required us to evaluate these cases in our study. Cataract was the most common cause of bilateral blindness (26.9%) and the second most common cause of monocular blindness (19%) in our study. This finding was similar to those in developing countries. A rate of 23.4% was reported in Ethiopia, 36.5% in Nigeria, 19.2% in Mali, and 34.9% in Mongolia.^10,16,17,18^ Based on our observations, patients present very late to ophthalmology clinics due to the lack of a publicly funded healthcare system in Somalia, resulting in them having to pay the full cost of cataract surgery themselves, and the low income in the general population. These reasons may explain why cataract is the most common cause of bilateral blindness.

In our study, diabetic retinopathy emerged as another common cause of blindness. We believe factors that may increase the impact of diabetic retinopathy as a cause of blindness in Somalia include the lack of intraocular injection therapy for diabetic macular edema, the inability to diagnose and treat with methods such as fundus fluorescein angiography and argon laser photocoagulation, as well as patients’ systemic comorbidities such as hypertension and kidney failure, and poor blood glucose control.

Another cause of blindness resulting from retinal damage in our study was age-related macular degeneration (6.6% unilateral, 9.7% bilateral). Blindness due to age-related macular degeneration and diabetic retinopathy is on the rise in both developed and developing countries. In Turkey, Özen Tunay et al.^19^ determined in their study of the geriatric age group that age-related macular degeneration was the most common cause of low vision in presenile and senile groups. As development continues and the average lifespan becomes longer in Somalia, we believe there will be an increase in vision loss associated with retinal diseases in the near future.

Glaucoma was found to be the third most common cause of bilateral blindness (15.4%). Glaucoma is a common cause of blindness in both developed and developing countries.^20,21^ In previous studies, blindness due to glaucoma was reported at rates of 7.1% in Pakistan, 15.4% in Sweden, 7.7% in China, 5% in Iceland, and 17% in Saudi Arabia.^3,21,22,23,24^

There are no previous studies reporting the prevalence of glaucoma in the Somali population. However, according to our clinical observations, glaucoma patients tend to present to the outpatient clinic at a late stage. We have also observed that awareness of glaucoma is low. Insufficient access to medicines (largely due to financial incapacity) and low awareness among patients are thought to contribute to blindness caused by glaucoma.

Although this study provides data regarding monocular and bilateral blindness from all regions of Somalia, it may not be able to determine the actual prevalence due to its hospital-centered design. We believe that society-based studies are necessary to establish the exact prevalence.

## Conclusion

Trauma is the most important cause of blindness due to national security conditions. The lack of a national healthcare system in Somalia is a notable factor in patients’ late presentation to healthcare clinics for their medical problems and increases the rate of blindness. Considering that 80% of blindness is preventable and treatable according to WHO data, primary healthcare services should be established in Somalia and these centers should be encouraged to conduct screening programs. Public hospitals with free service should be established to reduce the growing problem of blindness. These centers should include treatment and follow-up clinics for low-income patients with diseases such as cataract, glaucoma, and diabetic retinopathy.

## Figures and Tables

**Table 1:**
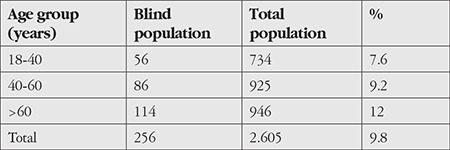
Blindness distribution by age

**Table 2:**
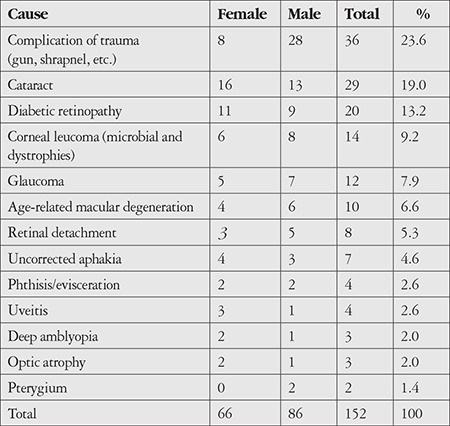
Causes of unilateral blindness

**Table 3:**
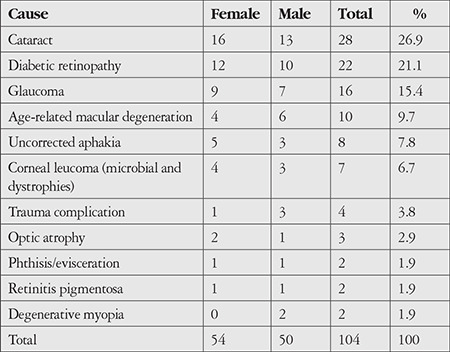
Causes of bilateral blindness
